# A Rare Case of Acute Post-cataract Surgery Endophthalmitis Associated With Achromobacter xylosoxidans

**DOI:** 10.7759/cureus.56527

**Published:** 2024-03-20

**Authors:** Tai Wan Dien, Nor Fariza Ngah

**Affiliations:** 1 Ophthalmology, Hospital Shah Alam, Selangor, MYS

**Keywords:** endophthalmitis, cataract, pars plana vitrectomy, intravitreal antibiotic, achromobacter xylosoxidans

## Abstract

*Achromobacter xylosoxidans* is rarely reported as a causative agent of post-cataract surgery endophthalmitis. Here, we present a case where timely surgical intervention preserved the patient's vision. A 68-year-old man presented with clinical signs of endophthalmitis in his right eye three days after uneventful cataract surgery. He was initially treated with intravitreal, topical, and systemic antibiotics.

After starting intravitreal, topical, and systemic antibiotics, his condition deteriorated on the second day of treatment. A prompt pars plana vitrectomy (PPV) with the removal of the posterior chamber intraocular lens (PCIOL) was performed. Culture from the intravitreal tapping yielded *A. xylosoxidans*, which was sensitive to ceftazidime and piperacillin. His condition was better post-PPV, and the infection was under control until day 10 post-first PPV. There was a recrudescence of infection with a recurrence of hypopyon and loculations detected on B-scan ultrasound. A second PPV with the complete removal of the lens capsule was performed. One month later, his right eye was quiet without inflammation, with a best-corrected vision of 20/30.

*A. xylosoxidans* is a rare but serious pathogen of endophthalmitis that often necessitates multiple surgical interventions. Although it may not initially respond to intravitreal injections and vitrectomy, appropriate treatment, such as the removal of the intraocular lens and capsulectomy, can still result in favorable visual outcomes.

## Introduction

Postoperative endophthalmitis is defined as intraocular inflammation caused by an infective process following intraocular surgery. Acute postoperative endophthalmitis presents within two weeks of surgery, whereas chronic post-operative endophthalmitis presents weeks or months after eye surgery [1]. Common organisms associated with acute post-cataract endophthalmitis include *Staphylococcus epidermidis*, *Staphylococcus aureus*, *Streptococcus pneumoniae*, and *Pseudomonas aeruginosa* [1].

*Achromobacter xylosoxidans* was initially described by Yabuuchi and Ohyama in 1971 as an aerobe, oxidase-positive, and motile Gram-negative rod [2,3]. It is commonly found in the respiratory tract. Immunocompromised individuals, such as those with underlying conditions like diabetes mellitus or those undergoing invasive procedures, are more susceptible to infections caused by this bacterium. *A. xylosoxidans* is a rare bacterium that causes endophthalmitis. It is often mistaken for *P. aeruginosa* due to similar presentations, and misidentification can occur, leading to delayed or inappropriate treatment [4]. Studies have shown that keratitis associated with *A. xylosidans* is often resistant to conventional antibiotics [5,6]. In endophthalmitis, this organism creates a biofilm to survive in a toxic environment, suggesting that intravitreal antibiotic treatment is less effective than the surgical approach. Effective surgery to curb the recurrence of this infection involves the removal of the intraocular lens and the entire lens capsule [7].

We report here the clinical features, predisposing factors, and treatment challenges of this rare cause of post-operative endophthalmitis.

This article was previously presented as a meeting poster at the 12th Conjoint Ophthalmology Scientific Conference (COSC) on September 15-17, 2023.

## Case presentation

A 68-year-old man with underlying well-controlled type II diabetes mellitus and hypertension underwent an uneventful phacoemulsification of the right eye with posterior chamber intraocular lens (PCIOL) implantation under local anesthesia. There was no significant past ocular history. On postoperative day 3, he presented with a painful red eye and reduced vision.

**Figure 1:**
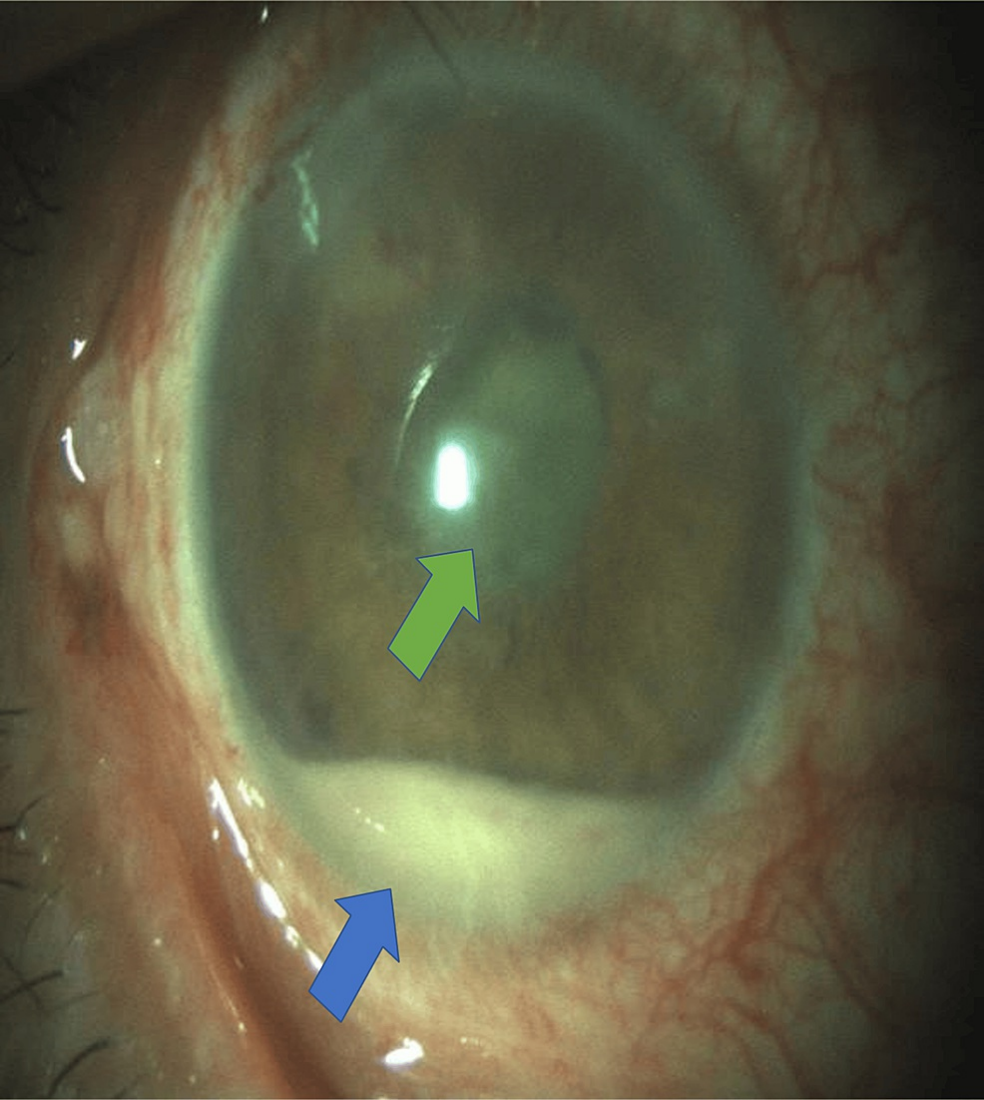
Right eye showed generalized conjunctiva injection, hypopyon (blue arrow), fibrin covering the pupil (green arrow) and posterior synechiae from 12 till 8 o’clock of pupil.

**Figure 2:**
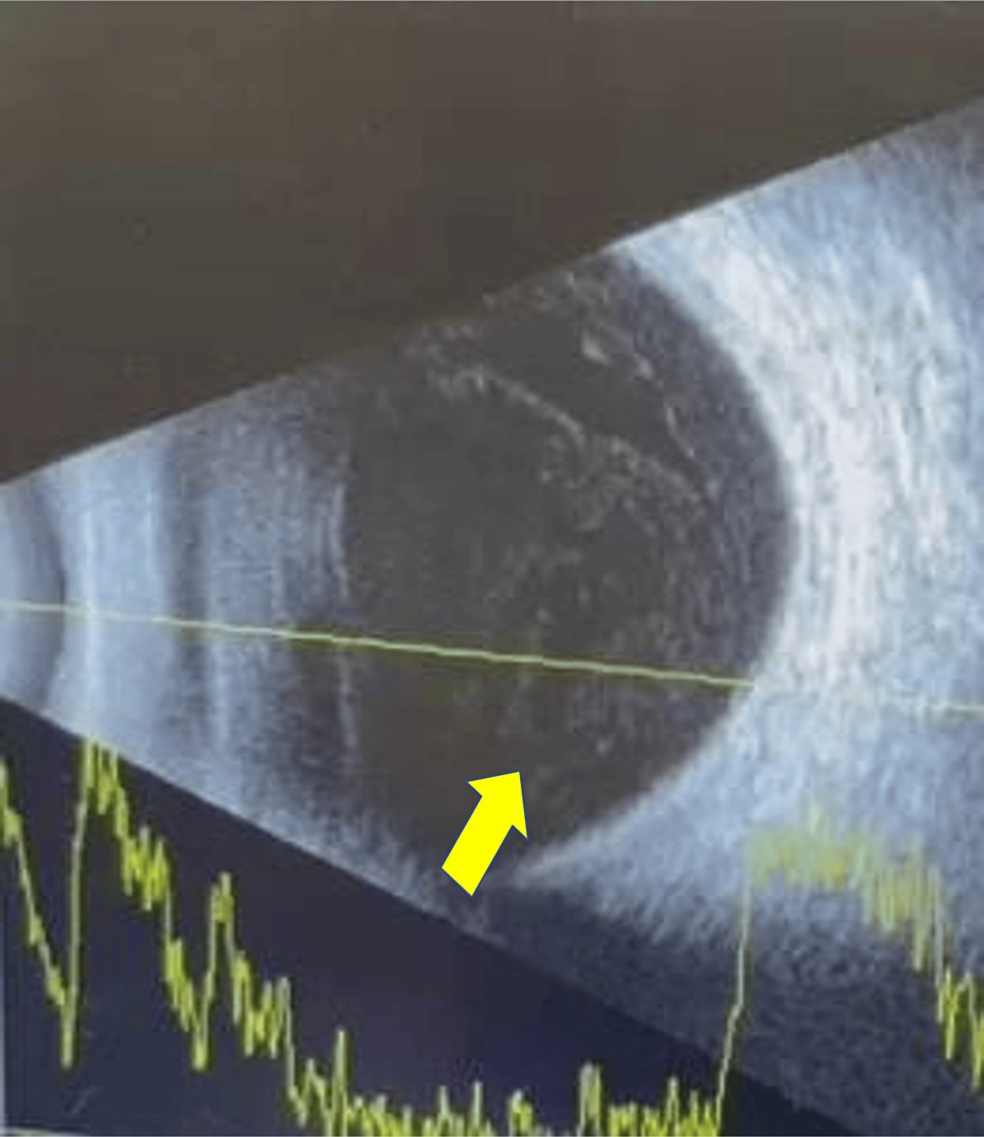
B-scan ultrasound of right eye with gain of 110 decibels showed heterogenous opacity with loculations (yellow arrow). The retina was flat.

Visual acuity in the right eye (Figure 1) was 20/100. Ocular examinations of the right eye revealed generalized conjunctiva injection. Paracentesis and the main wound were tight with a negative Siedel's test. The anterior chamber showed 4+ aqueous cells and flare associated with fibrin, which was covering the pupil, >1 mm hypopyon level, and posterior synechiae from 12 to eight o’clock of the pupil. The posterior segment showed dense vitreous. B-scan ultrasound of the right eye (Figure 2) showed heterogeneous opacity with loculations. Systemically, his general health condition was good, and his vital signs were normal. There were no signs of a primary source of infection. A blood investigation showed a normal level of white blood cell count.

Given the severity and acute presentation, he was treated as having right-eye acute postoperative endophthalmitis. He was immediately treated within an hour of presentation, which included vitreous sampling, intravitreal ceftazidime 2 mg/0.1 mL, vancomycin 2 mg/0.1 mL, systemic ciprofloxacin 750 mg BD [1], and intensive topical eyedrops ceftazidime 5%, gentamicin 0.9%, vancomycin 5%, and dexamethasone 0.1%.

Despite standard treatment for postoperative endophthalmitis, his condition deteriorated on day two post-treatment. Repeated intravitreal ceftazidime 2 mg/0.1 ml and vancomycin 2 mg/0.1 ml were performed. However, there was a poor improvement. He was referred for an early vitreoretinal intervention. On day 3 of the infection, he had a right pars plana vitrectomy (PPV) with the removal of PCIOL. The vitreous sample yielded a Gram-negative rod, and the culture and sensitivity revealed *A. xylosoxidans*, which was sensitive to ceftazidime and piperacillin. 

His condition improved after PPV, and the infection was under control until day 10 of PPV; there was a recurrence of hypopyon in the anterior chamber. The patient was again referred for a second PPV, and the complete removal of the lens capsule was done on the same day of referral.

After the second PPV, his condition improved with topical antibiotics and an anti-inflammatory agent. During the last review, at one month post-second PPV, his best corrected visual on the right eye was 20/30. We have scheduled a secondary IOL for him at a later date.

## Discussion

The common causative agents for acute postoperative endophthalmitis are *Staphylococcus epidermidis*, *Staphylococcus aureus*, *Streptococcus pneumoniae*, *Pseudomonas aeruginosa*, Aspergillus species, and Fusarium species, whereas for chronic postoperative endophthalmitis they are *Propionibacterium acne*, Corynebacterium species, and fungi [1]. A. xylosidans-causing endophthalmitis is rare. It is a Gram-negative motile rod, which is difficult to distinguish from Pseudomonas species in the laboratory and can lead to misidentification and inappropriate treatment [8].

*A. xylosoxidans* is a common fluid contaminant [9]. This organism is also part of the normal flora of the ear and gastrointestinal tract. However, it plays an important role in opportunistic nosocomial infection, especially among immunocompromised hosts, where it can complicate pneumonia, urinary tract infection, sepsis, and meningitis [10].

In 1977, Holmes et al. described the first *A. xylosoxidans*-related ocular infection, which they discovered in an infected orbit following a wound injury [11]. Reddy et al. reported the majority of the ocular manifestations to include corneal keratitis and endophthalmitis [12]. Despite the limited number of reports, this organism was found to cause serious and recalcitrant postoperative endophthalmitis. It is more commonly reported to cause chronic [4,8,13-17] postoperative endophthalmitis than acute postoperative endophthalmitis [4,7,10,18].

Despite the aggressiveness of systemic and topical antibiotics, our patient’s condition did not improve. He then underwent PPV and the removal of PCIOL three days after the acute presentation. His condition again deteriorated 10 days after the first PPV and required a second PPV with capsular bag removal. The special characteristic of this organism is that it can create a biofilm to survive in a toxic environment [19]. Hence, we believe that PPV alone is not enough to resolve this condition, but the removal of the IOL and lens capsule is ultimately critical to prevent recurrence and achieve resolution of the infection, as demonstrated by currently available data [4,7,10,18].

Previous studies highlight some unique challenges in the management of this organism. A case series reported by Villegas et al. had four different cases that presented as endophthalmitis from as early as day one postoperatively to four months postoperatively. Two cases successfully ceased the infection with early PPV and complete removal of the capsular bag, whereas another two cases failed with the initial "non-capsular bag removal approach" and eventually had the infection ceased with PPV and capsular bag removal. Villegas et al. have depicted histopathological evidence of the microorganism at the inner surface of the anterior and posterior capsules [4]. Donlan and Costerton reported that *A. xylosidans* create a biofilm to survive in a toxic environment, suggesting that complete intraocular lens and lens removal are essential for preventing *A. xylosidans* infection [19]. Park et al. reported a case series of patients with postoperative endophthalmitis who received a mean number of 10 intravitreal antibiotic injections and three vitrectomies, of which their recurrence only ceased after en-bloc removal of the intraocular lens and lens capsule [7]. In terms of antibiotics, the organism is susceptible to extended-spectrum penicillins (namely piperacillin, carbenicillin, and ticarcillin), ceftazidime, imipenem, and trimethoprim-sulfamethoxazole. Besides, it is found to be resistant to aminoglycosides, some fluoroquinolones, and first-generation cephalosporins [12]. It is believed that the molecular structure of ceftazidime can provide more stability to beta-lactamases than the older generation of cephalosporin [4]. Hence, ceftazidime and amikacin are antibiotics of choice for the management of ocular infections caused by this organism [12]. Our antibiotic susceptibility test showed that this organism is sensitive to ceftazidime and piperacillin.

Interestingly, a recent small case series by Tae et al. was the first to suggest that the removal of the IOL and lens capsule may be unnecessary, as all their cases were resolved with early, immediate PPV and empirical antibiotics [18].

Finally, most cases are resolved after surgical intervention. Villegas et al. reported a case that eventually required enucleation due to a painful blind eye with severe inflammation [4]. Otherwise, the majority of the patients were left aphakic in the current available data. There was one patient who underwent six times PPV and 23 intravitreal injections, ultimately had a scleral fixated IOL implanted into the eye, and was able to achieve a final visual acuity of 20/30 [7].

## Conclusions

In conclusion, *A. xylosoxidans* is a rare but resistant pathogen of endophthalmitis that necessitates multiple surgical interventions. It is essential to recognize the varying degrees of severity among the different causative agents of endophthalmitis for appropriate management and prognosis. *A. xylosoxidans* may not initially respond to intravitreal injections and vitrectomy, but it can still result in favorable visual outcomes with appropriate treatment, such as the removal of the intraocular lens and capsulectomy.
